# Long-Term Noninvasive Genetic Monitoring Guides Recovery of the Endangered Columbia Basin Pygmy Rabbits (*Brachylagus idahoensis*)

**DOI:** 10.3390/genes16080956

**Published:** 2025-08-13

**Authors:** Stacey A. Nerkowski, Paul A. Hohenlohe, Janet L. Rachlow, Kenneth I. Warheit, Jonathan A. Gallie, Lisette P. Waits

**Affiliations:** 1 Department of Fish and Wildlife Sciences, University of Idaho, Moscow, ID 83844, USA; stacey_nerkowski@fws.gov (S.A.N.); jrachlow@uidaho.edu (J.L.R.); 2 United States Fish and Wildlife Service, Northeast Fishery Center, Lamar, PA 16848, USA; 3 Department of Biological Sciences, University of Idaho, Moscow, ID 83844, USA; hohenlohe@uidaho.edu; 4 Washington Department of Fish and Wildlife, Olympia, WA 98501, USA; kenneth.warheit@dfw.wa.gov; 5 Washington Department of Fish and Wildlife, Wenatchee, WA 98801, USA; j.gallie@dfw.wa.gov

**Keywords:** genetic monitoring, noninvasive genetic sampling, pygmy rabbits, *Brachylagus idahoensis*, native populations, wildlife

## Abstract

**Background/Objectives:** Loss and fragmentation of habitat from agricultural conversion led to the near extirpation of the pygmy rabbit (*Brachylagus idahoensis* Merriam, 1891) population in the Columbia Basin (CB) of Washington, USA. Recovery efforts began in 2002 and included captive breeding, translocations from other regions for genetic rescue, and reintroduction into native habitat in three sites: Sagebrush Flat (SBF), Beezley Hills (BH), and Chester Butte (CHB). **Methods:** We used noninvasive and invasive genetic sampling to evaluate demographic and population genetic parameters on three translocated populations of pygmy rabbits over eight years (2011–2020). For each population, our goal was to use fecal DNA sampling and 19 microsatellite loci to monitor spatial distribution, apparent survival rates, genetic diversity, reproduction, effective population size, and the persistence of CB ancestry. Over the course of this study, 1978 rabbits were reintroduced as part of a cooperative conservation effort between state and federal agencies. **Results:** Through winter and summer monitoring surveys, we detected 168 released rabbits and 420 wild-born rabbits in SBF, 13 released rabbits and 2 wild-born in BH, and 16 released rabbits in CHB. Observed heterozygosity (H_o_) values ranged from 0.62–0.84 (SBF), 0.59–0.80 (BH), and 0.73–0.77 (CHB). Allelic richness (AR) ranged from 4.67–5.35 (SBF), 3.71–5.41 (BH), and 3.69–4.65 (CHB). Effective population (N_e_) within SBF varied from 12.3 (2012) to 44.3 (2017). CB ancestry persisted in all three wild populations, ranging from 15 to 27%. CB ancestry persisted in 99% of wild-born juveniles identified in SBF. Apparent survival of juvenile rabbits differed across years (1–39%) and was positively associated with release date, release weight, and genetic diversity. Survival of adults (0–43%) was positively influenced by release day, with some evidence that genetic diversity also positively influenced adult apparent survival. **Conclusions:** Noninvasive genetic sampling has proven to be an effective and efficient tool in monitoring this reintroduced population, assessing both demographic and genetic factors. This data has helped managers address the goals of the Columbia Basin recovery program of establishing multiple sustainable wild populations within the sagebrush steppe habitat of Washington.

## 1. Introduction

Loss of biodiversity is one of the most important environmental problems facing the world today [1]. Rapid human population growth, environmental change, and habitat fragmentation all pose ever-greater threats to biodiversity and highlight the need for increasingly aggressive conservation efforts [2]. One important conservation tool is genetic monitoring [3,4,5,6]. Genetic monitoring studies have been used to address many conservation issues, including population abundance [7,8,9,10], population assignements and population structure [11,12,13,14], parentage analysis [15,16], genetic diversity levels [17,18,19] and population bottlenecks [20,21,22]. Noninvasive genetic sampling has become a common method for sample collection in many genetic monitoring studies. Noninvasive genetic sampling allows researchers to monitor populations through the collection of feces, hair, saliva, feathers, or any other biological material left behind by an animal without capturing, disturbing, or even observing individuals [23,24,25,26].

Endangered species and isolated populations typically face genetic threats such as loss of genetic variation and inbreeding that can ultimately lower the fitness of the individual and population [27]. Genetic rescue, translocations into a small population to alleviate these issues, has the potential to be one of the most powerful means to conserve small and declining populations [28,29,30,31]. A major concern with genetic rescue is that gene flow can decrease fitness through outbreeding depression, potentially increasing the risk of extinction [29], although recent cases demonstrate its potential to aid in population persistence. Genetic rescue has increased genetic variation and resulted in population recovery for a variety of terrestrial wildlife species including Mexican wolves (*Canis lupis baileyi* [32]), Florida panthers (*Puma concolor coryi* [33]), greater prairie chicken (*Tympanuchus cupido* [34]), arctic fox (*Vulpes lagopus* [35]), and bighorn sheep (*Ovis canadensis* [36]). However, monitoring for potential negative consequences of genetic rescue is crucial in assessing the outcome of the genetic rescue for the population [37].

**Figure 1:**
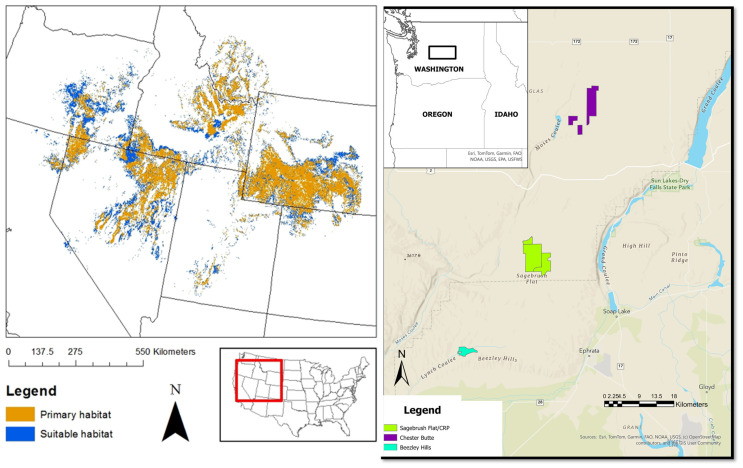
(**Left**) Pygmy rabbit distribution across the western United States Taken from Smith et al. (46). (**Right**) Geographic location of the reintroduced Columbia Basin pygmy rabbit (*Brachylagus idahoensis*) populations in Washington, USA, and locations of Sagebrush Flat, Beezley Hills, and Chester Butte recovery areas.

Here we present an 8-year study using traditional tissue sampling and noninvasive fecal DNA sampling to monitor the world’s smallest rabbit, the pygmy rabbit (*Brachylagus idahoensis*). Pygmy rabbit populations are found in sagebrush (*Artemisia* ssp.) habitats across the western United States, including portions of the states of Wyoming, Utah, Nevada, Oregon, California, Montana, Colorado, and Idaho. A small, disjunct population occurs within the Columbia Basin (CB) of central Washington (Figure 1).

The CB population in Washington has been spatially and genetically isolated for at least 10,000 years and has been present in the area for nearly 100,000 years [38,39]. The CB pygmy rabbits are considered a distinct population segment, the smallest division of a species warranted protection under the Endangered Species Act and were state-listed (Washington) in 1993, and federally emergency listed (Endangered Species Act) in 2001, with a final ruling in 2003 [40,41,42,43]. At the time of federal listing, the population included fewer than 30 individuals in the wild, and the geographic distribution in Washington was reduced from 6 populations in five counties in the 1990s to a single population at Sagebrush Flat Wildlife Area (SBF) in Douglas County [40,41,42,43].

In an attempt to save the population from extinction, the last remaining 16 rabbits were captured and brought into captivity in 2001 to establish a captive breeding population to support future reintroduction efforts [43]. Decreased reproductive success in captivity and low genetic diversity suggested that the CB population was experiencing inbreeding depression [39,44]. To counteract potential inbreeding depression and provide for genetic rescue, four pygmy rabbits from Idaho were introduced into the captive breeding program in 2003 [43,45]. Breeding was carefully managed to prevent inundating the captive CB population with Idaho genetic variation and to preserve unique CB ancestry while maintaining genetic health [44].

With the main goal of the CB Recovery Program to establish a sustainable wild population, the captive breeding program ended in 2011 and transitioned to semi-wild, onsite breeding enclosures [45]. To provide further genetic rescue and the necessary numbers needed for release, 111 wild pygmy rabbits were translocated from Oregon, Nevada, Utah, and Wyoming, and were kept in the same large enclosures to encourage interbreeding. Since the first releases in 2011 onto Sagebrush Flats (SBF, Figure 1), a total of 1,978 pygmy rabbits have been released in three recovery areas [46]. Monitoring of released individuals, spatial expansion, mixed ancestries, population size and reproduction in the wild is crucial to the overall goal of a sustainable wild population. Additionally, in summer 2018, two new reintroduction sites were established in the Beezley Hills (BH) and Chester Butte (CHB) Recovery Areas (Figure 1).

Noninvasive genetic sampling of fecal pellets has become a valuable method for monitoring the reintroduced CB pygmy rabbit populations [47,48]. DeMay et al. [49] used microsatellite loci to perform parentage analyses to assess the influence of ancestry, population density, and genetic diversity on reproduction within the breeding enclosures. A decline in male reproductive output was detected as population density increased and genetic diversity decreased. Males with >50% Columbia Basin ancestry had higher reproductive output whereas males of northern Utah/Wyoming ancestry had lower reproductive output. Female reproductive output decreased with Nevada/Oregon ancestry [49]. This information indicated that ancestry plays an important role in reproductive fitness in the captive population and is likely important in the wild/released populations.

The goals of this study were to combine data from 2012 to 2020 to (1) assess spatial distribution of wild populations, (2) assess demographics including sex ratio, minimum census population size and density of the wild population, (3) estimate genetic diversity, effective population size and persistence of CB ancestry of wild populations, (4) assess apparent survival of released pygmy rabbits to determine which genetic and/or demographic factors influence survival (Table 1). We used these data to test hypotheses and make conservation recommendations.

We predicted that CB ancestry would be maintained in the wild population because juveniles with higher CB ancestry were retained as breeders in the captive population. We expected a decrease in heterozygosity, over the 8 years in both wild and in enclosure populations because of the limited number of founders, and we expected an N_e_ < 100. We also predicted that apparent survival rates of juveniles would be positively influenced by year, release weight, and release day, and that apparent survival of adults would be positively influenced by release day and heterozygosity [48,50]. We expected that apparent survival rates would increase for rabbits released later in the year because they were vulnerable to predation for a shorter amount of time before winter surveys. We also expected that older juveniles would have a higher probability of survival because they had more time in the breeding enclosures with high-quality food and protection from predators and could achieve better body condition prior to being released compared to those released at younger ages [51].

## 2. Materials and Methods

### 2.1. Study Area

**Figure 2:**
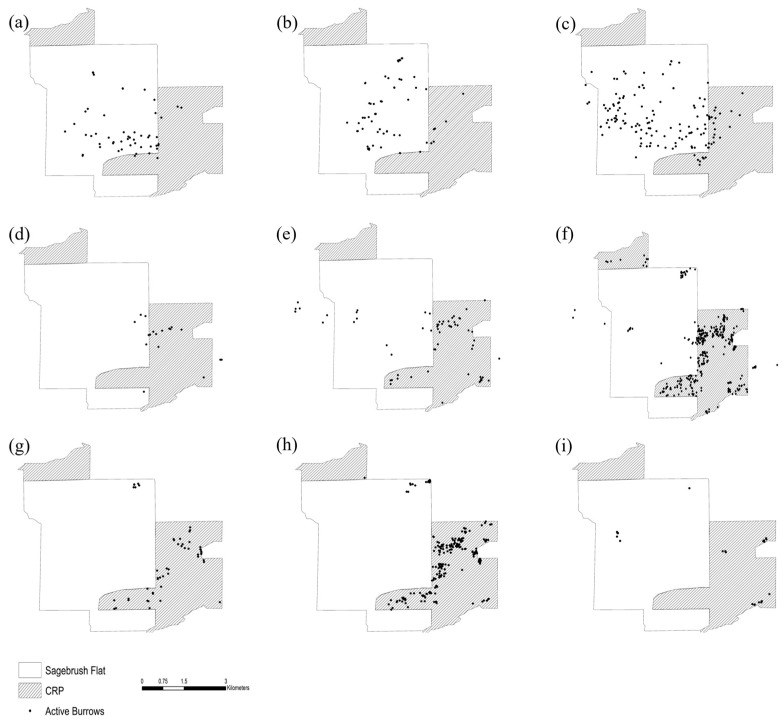
Location of Sagebrush Flat (SBF) wildlife area in Washington, USA, Conservation Reserve Program habitat (CRP), and active pygmy rabbit (*B. idahoensis*) burrows (•) identified during winter monitoring surveys during (**a**) winter 2012–2013, (**b**) winter 2013–2014, (**c**) winter 2014–2015, (**d**) winter 2015–2016, (**e**) winter 2016–2017, (**f**) winter 2017–2018, (**g**) summer 2018, (**h**) winter 2018–2019, and (**i**) winter 2019–2020. Area to the left of SBF represents private land.

The study area for this project included three study sites in central Washington, USA: Sagebrush Flats (SBF; 1514 ha; Latitude/Longitude: 47.667, −119.684), and Chester Butte (CHB; 893 ha; Latitude/Longitude: 47.711, −119.549) in Douglas County, and Beezley Hills (BH; 83 ha; Latitude/Longitude: 47.325, −119.836) in Grant County (Figure 1). All study sites were located on the Columbia Plateau Province (Crab Creek sub-basin). SBF and CHB are separate units of the larger Sagebrush Flat Wildlife Area (SFWA) managed by the Washington Department of Fish and Wildlife (WDFW). The SFWA is managed specifically for endangered and threatened species including pygmy rabbits, greater sage-grouse (*Centrocercus urophasianus*), and sharp-tailed grouse [52]. BH was a combination of private land and land owned and managed by The Nature Conservancy [46]. These sites were characterized by dense sagebrush (*Artemisia ssp*.) and deep soils, and SBF also contained mima mounds, micro-topographic features that are composed of loose, unstratified sediment that supports relatively dense sagebrush, grasses and forbs [52,53]. All sites were surrounded by state, federal, and private lands, with a land cover mosaic of sagebrush steppe and wheat fields. The SBF Unit was also surrounded by Conservation Reserve Program (CRP) lands, which are agricultural fields that were revegetated with sagebrush-steppe flora in the mid-1990s [52] (Figure 2). Predators of pygmy rabbits within the study area include badgers (*Taxidea taxus*), long-tailed weasels (*Mustela frenata*), coyotes (*Canis latrans*), short-eared owls (*Asio flammeus*), and several other raptor species. Temperatures (30-year average) ranged from an average minimum of −6 °C in December to an average maximum of 31.2 °C in July [54]. This semi-arid environment averages about 20.3 cm of annual precipitation, over half of which falls as snow [52,54].

### 2.2. Release Efforts

Semi-wild breeding enclosures were initially established in SBF (2012 breeding season) where two large, predator-resistant enclosures (2.3 and 4.4 ha) were constructed [43]. For the 2013 breeding season, a third enclosure (2.2 ha) was added at a second site, 25 km to the north. For the 2014 breeding season, a fourth enclosure (3.8 ha) was constructed 17 km southeast of the first two [55]. Each enclosure’s fences were buried approximately 45 cm into the ground and featured a ‘floppy top’ design to protect against terrestrial predators, while protective netting over pygmy rabbit burrow systems and bird spikes installed on fence posts discouraged avian predators [55]. Although the enclosures were designed to keep terrestrial predators out, weasels were removed from the enclosures across the years and avian predations did occur in unprotected matrix between covered burrow systems. From 2011–2016, artificial feeding stations were set up in the large predator-resistant enclosures where greens lettuce/alfalfa were provided weekly, dry alfalfa and rabbit chow were provided ad libitum. Irrigation systems in all enclosures provided rabbits with water supply at each feeding station and irrigated vegetation on site. Artificial burrows (0.91 m long plastic black piping) were also constructed at each feeding station. In 2017, irrigation was discontinued, and the number of feeding stations were reduced to address increased levels of coccidia, and lettuce and alfalfa at feed stations was reduced to address increased weed production within the enclosures [56].

Juvenile pygmy rabbits were captured from breeding enclosures and released to the wild or kept for breeding during the 2012–2019 breeding seasons. Individuals were captured using Tomahawk live traps (Tomahawk Live Trap Co., Hazelhurst, WI, USA) set at burrow entrances or known rabbit trails and covered with burlap to minimize stress on the individual. In large enclosures, juveniles were captured from open-ended artificial burrows (buried 10-cm diameter drainage tubes, approximately 1-m long) using a modified version of a plumber snake with a tennis ball on one end to easily push juveniles into a cloth handling bag on the opposite end. Starting in 2017, net panels were designed to capture juveniles that were herded toward a wall of net panels, enabling researchers to flush rabbits to an area. Captures and releases occurred from late April to late September. All capture and handling methods were approved by the University of Idaho Animal Care and Use Committee (Protocol 2012-23, 2017-25, and 2020-13), were consistent with the standard for use of wild mammals in research established by the American Society of Mammologist [57] and were performed in accordance with applicable laws governing the use of endangered species.

Initially, release efforts focused primarily on juveniles (Table 2). In 2014, to reduce overcrowding within the breeding enclosures, we released adults as well. From 2012 to 2014, all released individuals were translocated to SBF. In 2015, we released 153 individuals (149 juveniles and 4 adults) into SBF, but to establish a second population, we released 420 individuals (369 juveniles and 51 adults) into the BH recovery area (Table 3). In 2016, we released all individuals into SBF. Although 37 kits were released into the BH area in 2017, a wildfire burned 119 km^2^ of sagebrush-steppe habitat in June 2017 and destroyed the BH enclosure (Sutherland Canyon fire). In a second attempt by managers to establish a population at BH, and a new reintroduced population in the CHB recovery area, all rabbits from 2018 to 2020 were released to one of these areas. We released rabbits into the BH and CHB release areas following a soft release protocol. Rabbits were placed into 0.40-ha circular pens with temporary fencing made of chicken wire. These release pens were left in place until the end of winter, but in some years, snow accumulation allowed rabbits to move in and out of the release pens during winter.

All juveniles and adults trapped in the enclosures were weighed, sexed, and treated for parasites with Advantage II kitten formula (BayerDVM, Shawnee Mission, KS, USA). We collected a 2 mm skin biopsy from the ear, which was stored in 95% ethanol and frozen at −20 °C until laboratory analysis could be performed. Juveniles that were retained as breeders typically contained higher levels of CB ancestry ($\bar{X}=29.1\%\pm \;$13.86%). All individuals retained for breeding were microchipped (Avid Identification Systems, Inc., Norco, CA, USA). Individuals were also swapped among the enclosures to reduce breeding among related individuals and increase the genetic diversity of future breeding.

Individuals released at SBF followed mostly hard-release methods that did not include acclimation pens. Rabbits were released into artificial burrow systems in shrubstep habitat across 2–6 release areas (17–37 release sites per area) as described in DeMay et al. [48]. Augmentation in the SBF population ended in 2016; beginning in summer 2017, juveniles were placed in temporary release pens (0.40 ha) to increase survivorship and limit dispersal distances at the BH and CHB recovery areas. These release pens were considered a soft release protocol, allowing for acclimatization to the new habitat, in which the pens were breached during winter months. No more than 10 juveniles were placed into a release pen.

Because of the limited number of individuals in the enclosures, in summer (2018–2019), we translocated wild-born juveniles from the SBF population to breeding enclosures and release pens in the BH and CHB release areas. Wild trapping protocols were the same as the enclosure trapping protocols described above. All wild adult rabbits caught were weighed, sexed, and a genetic sample was obtained through a 3 mm ear biopsy before being released back into the burrow where they were trapped.

### 2.3. Winter Surveys

We conducted winter surveys each year following releases to locate active burrows and collect fecal pellets for genetic analysis [48]. Ideally, surveys were conducted under fresh snow conditions, but in years with relatively low snowfall, some surveys were performed with no or minimal snow cover. We performed surveys of 35–50 m wide belt transects by foot, prioritizing release sites and areas with active burrows from previous years and then expanding outward. When snow was present, we followed rabbit tracks and trails to active burrows. From 2012 to 2017, all winter surveys were conducted at SBF. During 2018–2020, winter surveys were conducted at SBF, BH, and CHB release areas. The area surveyed each year depended on the availability of WDFW personnel and volunteers, and accessibility to survey areas (Table 2 and Table 3). Total area surveyed was calculated by the global positioning system (GPS) track files from each surveyor, or if track files were unavailable, the overall area was estimated by a polygon in ArcMap (ESRI, Redlands, CA, USA). At each active burrow, the GPS coordinates, number of entrances, activity level (high, medium, and low based on pellet numbers and distribution), and visual confirmation of a rabbit were recorded. A minimum of three fecal pellets were collected to ensure an adequate amount of DNA for genetic analysis [58]. Fecal pellets were collected from a single, distinct pile of pellets to increase the probability that the sample represented a single individual. Fecal samples were stored in paper envelopes, desiccated with silica gel beads, and kept a room temperature (~23 °C) until laboratory analysis could be performed.

### 2.4. Summer Surveys

Beginning in 2018, we initiated summer monitoring in SBF, and monitoring started in 2019 for BH and CHB (Table 2 and Table 3). The goal of summer monitoring was to detect kits and evaluate the status of rabbits in prioritized areas near release pens or active burrows from the previous winter. At each active burrow, we used the same protocol described above for winter monitoring. Since juveniles and adults are present during the summer, multiple fecal samples were often collected from the same burrow system. Juvenile pellets were identified as pellets ≤ 2.5 mm in diameter, whereas adult pellets were typically 4–5 mm in diameter. Fecal pellets were stored as described above.

### 2.5. Laboratory Methods

DNA was extracted from tissue samples collected from rabbits using Qiagen DNeasy blood and tissue kits (Qiagen Inc., Valencia, CA, USA) following the methods described in DeMay et al. [55]. We amplified extracted DNA in duplicate across 19 microsatellite loci (18 autosomal loci and 1 Y-chromosome locus) within 3 polymerase chain reaction (PCR) multiplexes [55]. Samples were run on an Applied Biosystems 3130xl Genetic Analyzer (Applied Biosystems Inc., Foster City, CA, USA), and results were analyzed in Genemapper 5 (Applied Biosystems Inc.) and confirmed visually. Any unknown adult sample was compared to previously known individuals to determine if there was a match or if the individual was a new rabbit.

DNA of fecal pellets collected during winter and summer monitoring was extracted using the Qiagen QIAmp DNA Stool Mini Juvenile (Qiagen Inc., Valencia, CA, USA) in a laboratory dedicated to low-quantity DNA samples [58]. We performed species ID tests using a 294-bp fragment of the mitochondrial DNA cytochrome b gene following the protocols described in Adams et al. [58]. The species ID test was designed to distinguish between pygmy rabbits and sympatric cottontail species (*Sylvilagus nuttallii*, *S. audobonii*, *S. floridanus*). For the 2012–2013 and 2013–2014 surveys, all samples underwent a species ID test, but after further testing, it was determined that cottontail samples did not amplify or produce out-of-bin alleles at various microsatellite loci [11], which could successfully exclude these individuals without performing a species identification (ID) test. During 2014–2018, only samples that failed the microsatellite analysis were run on the species ID panel. In 2018, we reinstituted the species ID panel on all samples before analysis on the microsatellite panels due to declining numbers of pygmy rabbits. Any sample that failed to amplify on the species ID test or amplified as cottontail was excluded from the remainder of the analyses.

We initially amplified all samples that were confirmed pygmy rabbit in duplicate, on the first PCR multiplex consisting of 8 loci (A12, A124, A140, Sat7, Sat8, Sol08, Sol44, sex locus-Y05) following the protocols described in DeMay et al. [48]. Genotypes at these loci were then compared to the genotypes of known individuals to determine if there was a match, but also to screen out low-quality samples. Pellets had to amplify at ≥5 of the loci (excluding the sex loci) in the first to move on to the second multiplex consisting of 7 loci (A113, A121, A133, A2, D118, Sat5, and sex-locus Y05), and ≥4 loci were required (excluding the sex locus) to meet P(ID)sibs < 0.01 and verify a match from 2012 to 2017 [48]. In 2018, we used the 2nd and 3rd PCR multiplexes (5 autosomal loci—A128, A129, D103, D2, and 7LID3) in combination rather than the 1st PCR multiplex to increase statistical power in distinguishing individuals as the degree of relatedness among individuals increased. A minimum of 8 loci was required to meet P(ID)sibs < 0.01 and verify a match using multiplex 2 and 3 from 2018 to 2020. We ran pellet samples a minimum of four times and up to eight times to produce a consensus genotype. Two repeats of each allele were required to confirm a heterozygous genotype and three repeats to confirm a homozygous genotype [47]. Using the 12 loci, consensus genotypes were compared to one another to determine matching genotypes at multiple locations and matching to genotypes of previously released rabbits. Fecal samples that did not match a known rabbit were considered new wild born individuals and amplified for all remaining loci.

### 2.6. Analytical Methods

All tissue and fecal genotypes were added to a reference database, which also included morphological and demographic parameters on released and enclosure-born individuals. Fecal sample genotypes and unknown adult tissue genotypes were matched using GenAlEx 6.51 [59,60]. Matchings that contained 1 or 2 mismatches were further analyzed for human error or allelic dropout that resulted in the mismatches. We included locus A124 in the first multiplex for individual identity, but we removed it from all downstream analysis due to the high frequency of null alleles [48]. All parentage and population genetic analyses were conducted with the remaining 19 loci.

We analyzed all samples for parentage using a strict exclusion approach in Cervus 3.0.7 [61,62]. Parentage assignments that mismatched at 1–2 loci were once again examined for genotyping error, where a mismatch at a single locus, representing a single stepwise mutation, was accepted as a match. We used the program STRUCTURE 2.3.4 to assess ancestry based on the predefined groups identified previously—CB, Oregon/Idaho/Nevada, northern Utah/Wyoming, and southern Utah [63] genetic estimates for all individuals in this study, including wild-born individuals, released individuals, and enclosure individuals. STRUCTURE was run ten times with K = 4 under an assumption of admixture, correlated allele frequencies, and the LOCPRIOR model (prior information on the identified populations), with 100,000 cycles of burn-in (BURNIN = 100,000) and 500,000 Markov chain Monte Carlo samples (NUMREPS = 500,000). We estimated allele frequencies for each genetic cluster from individuals known by pedigree or capture records for each of the four predefined clusters and were used to estimate the CB ancestry for all non-founding individuals. Based on the STRUCTURE admix assignments to the CB predefined group for individuals known to be from predefined clusters other than the CB (admix assignments 0–4.89%), only individuals with estimates of ≥5% CB ancestry was identified as containing CB ancestry (Table 4 and Table 5).

We characterized genetic diversity and CB ancestry estimates for each winter survey year (Table 5). The wild population was defined as all new wild-born individuals, released individuals, and previously detected individuals sampled within a single year. We evaluated allelic richness (AR) using the R program hierfstat version 0.5-11 [64] and rarefied to a sample size of 5. Observed heterozygosity (H_o_) and unbiased expected heterozygosity (H_e_) were calculated using GenAlEx 6.5.1 [59,60]. H_e_ and AR were calculated only for sample sizes ≥5. Year-to-year and initial year-to-final year comparisons of H_o_, CB, and AR were evaluated using a Welch two-sample *t*-test in R [65]. Comparison of sex ratios from year to year were analyzed with two-sided Fisher’s exact test in R. We determined N_e_ for each winter survey year using the linkage disequilibrium model with random mating, minor allele frequency equal to 0.05, and 95% intervals in the parametric model in the program NeEstimator V2.1 [66] using the co-ancestry method [67] for the SBF populations only. We reported N_e_ estimates for sample sizes ≥ 7 because smaller sample sizes produced infinite estimates. Density estimates were based on the minimum count of rabbits identified each survey period/potential habitat (ha) within each recovery area (SBF = 1780 ha, BH = 83 ha, and CHB = 893 ha).

### 2.7. Apparent Survival Models

Apparent survival was defined as the detection of a released pygmy rabbit from fecal DNA collected during winter and/or summer surveys. Wild-born rabbits were not included in the apparent survival models because their life stage was unknown. In the SBF recovery area, we used logistic regression to assess juvenile and adult apparent survival, with winter/summer detection as the explanatory variable, as previously described [11]. A priori model sets were evaluated using Akaike’s Information Criteria corrected for small sample size (AICc), log-likelihood values, and model average parameter estimates with 85% confidence intervals [68] using R *AICcmodavg* [69]. We averaged parameter estimates across all the candidate models that included each given parameter. DeMay et al. [48] only evaluated adults released in 2014 because of the small number of adults released in 2012–2013, but our models set also included adults released in 2015 and 2016.

For apparent survival of adults, we included the explanatory variables release day, sex, release weight, homozygosity by loci (HL) calculated using the R package GENHET version 3.1 [70], and genetic estimate of CB ancestry derived from the protocols described above. Our candidate model set included all 30 possible combinations of the explanatory variables and the null model. Typically, before release, all juveniles were trapped and weighed, but in the case of released adults, weights were not always taken at time of release. The top model without release weight as an explanatory variable was compared to the top model including release weight for those individuals that had a recorded weight at time of release.

For apparent survival of juveniles, we included each combination of the explanatory variables with release year (categorical variable, 2012–2016). We used 2014 as a reference year, as in DeMay et al. [48], due to the large sample size. Our candidate model set included year (*p*-value = 2.2 e10^−16^) in each model and all possible remaining combinations, for a total of 32 models. For apparent survival in release pens, the same explanatory variables were used as in the juvenile model in SBF. The top model for release pen survival was compared to the top juvenile model to test for a difference in survival rates between the hard-release and soft-release approaches.

## 3. Results

### 3.1. Genetic Monitoring at Sagebrush Flat

Winter surveying efforts of the SBF area encompassed 5.89–24.28 km^2^, with an average of 12.70 km^2^ across the 8 years of surveys (Table 2). During 2012–2014, a common 6.7 km^2^ area was surveyed each year because burrows predominantly occurred in this area (Figure 2a–c), but in 2015, most pygmy rabbit habitat use shifted into the Conservation Reserve Program (CRP) land to the east and south of SBF (Figure 2d). Winter 2016–2017 had the greatest survey coverage (24.28 km^2^) because SBF and CRP fields were both surveyed. A decrease in the area surveyed occurred in 2017–2020, as pygmy rabbits occupied less spatial area.

**Figure 3:**
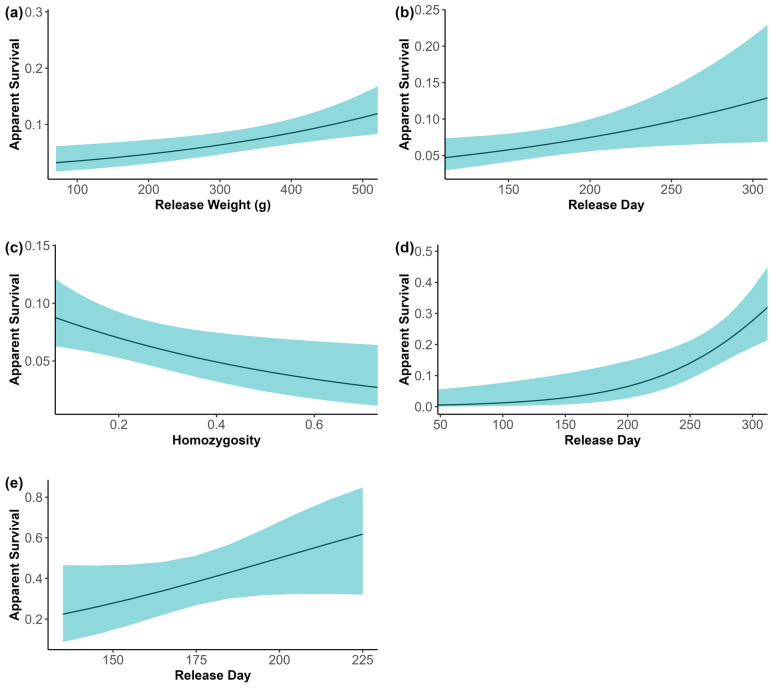
Predicted probabilities of apparent survival rates for pygmy rabbits (*B. idahoensis*) introduced into the central WA for significant variables in each model. (**a**) Juvenile survival rate by release weight for released juveniles in the Sagebrush Flat recover area (SBF), (**b**) juvenile survival rate by release day for released juveniles in SBF, (**c**) juvenile survival rate by homozygosity for released juveniles in SBF, (**d**) adult survival rate by release weight for released adults in SBF, (**e**) juvenile survival rate by release day for juveniles released into pens in the Beezley Hills and Chester Butte recovery areas. Predicted probabilities plots were generated from top models for juvenile and adult survival.

Successful species ID amplification ranged from 78–97% and was first implemented consistently starting in winter 2018–2019. Most of the pellets collected each year were identified as pygmy rabbit, with very few cottontail pellets collected (0–51%, $\bar{X}=10\%$) except in the winter 2019–2020 survey effort, when 51% of pellets collected were identified as cottontail. Of the pellets that were identified as pygmy rabbit, individual identity was successfully determined for 20–83% of the samples ($\bar{X}=60\%\;\pm \;20\%\;SE$). Years with lower success rates typically resulted from collection with minimal to no snow present and/or rain on snow events with frequent freezing and thawing. During 2012–2014, rabbits were spatially distributed primarily on the state-owned lands of SBF, and fewer than 10% of all active burrows identified were located on private CRP lands (Figure 2a–c). In winter 2014–2015, more active burrows (~28%) were located on the eastern and south-eastern border between SBF and CRP (Figure 2c). In winter 2015–2016, there was a substantial shift in the distribution, with ~20% of burrows located in the SBF area, and the remaining burrows (~80%) were found in CRP (Figure 2d). By winter 2016–2017, over 75% of all active burrows were located in CRP, 18.5% of burrows were located on private land to the west (Figure 2e), and 6.5% in SBF. Samples presumed to be pygmy rabbit, based on size, were also collected from a location approximately 16 km southeast of SBF, but the samples did not amplify on any genetic tests. The spatial distribution of rabbits exhibited in 2016 was also observed in winter 2017–2018 (Figure 3a), but with a decrease in the number of burrows on private land to the west. By winter 2018–2019, less than 6% of all burrows identified were located in SBF (Figure 3c), and by winter 2019–2020, all active burrows were located in CRP (Figure 3d). The summer 2018 survey also exhibited a similar spatial distribution as the winter 2018–2019 surveys, where burrows within SBF were limited to two small pockets in the west and north-east corner, and the remaining burrows were found in the CRP to the east and south (Figure 3b).

During 2012–2014, very few wild-born rabbits (3–16% of detected rabbits) were identified, and most individuals detected were released that year (77–96% of detected rabbits) (Table 2 and Table 4). Beginning in 2015, a higher proportion of wild-born rabbits (89–100% of those detected) were identified, with a smaller proportion of released individuals detected (6–8%). Only 1% (n = 25) of released or wild-born individuals were detected during a second year, and wild-born rabbits were approximately equally likely (5%) to be detected a second year as released rabbits (4%). Only one wild-born individual, identified in 2016 (0.1%), was detected in three consecutive winter surveys (2016–2018). Initially (2013–2015), the individuals detected in a subsequent year were released individuals, but as the number of wild-born individuals increased, detection of second-year individuals were primarily of wild-born descent (2016–2019). The highest detection of second-year wild-born individuals was in winter 2018–2019 (14 individuals—10% of rabbits detected that winter).

During 2012–2020, the number of rabbits per active burrow system averaged 0.74 ± 0.22 rabbits/burrow with a range of 0.33–1.00 (Table 4). The winter 2019–2020 survey produced the lowest number of rabbits/burrow system (0.33 rabbits/burrow), and the 2015–2016 survey produced the highest (1.00 rabbits/burrow), where every burrow found represented a new individual. A total of 67% of the years (6/9) fell above the mean, and 89% (8/9) were equal to or above 0.56 rabbits/burrow. Rabbits/burrow decreased during 2012–2014 but increased in 2015 (1.00) as rabbits shifted to CRP (Table 4). Density estimates varied year to year, ranging from 0.004 (2019–2020) to 0.09 (2017–2018), averaging 0.04 ± 0.03 for the SBF/CRP recovery area (Table 4).

The SBF population size fluctuated in numbers during 2012–2020. Since 2012, the minimum count of rabbits identified in winter surveys ranged from 8 to 158 (Table 2 and Table 4). During 2012–2016, the main augmentation to the SBF population occurred through reintroductions from the enclosure populations, with the number of released individuals ranging from 104 to 717 juveniles and 0 to 113 adults (Table 2), but during 2017–2020, no rabbits were released into the SBF area. During 2012–2014, the number of juveniles and adults released into SBF increased because of increased productivity within each of the enclosures. In 2014, to minimize the negative habitat effects resulting from many pygmy rabbits in the enclosure, most adults and juveniles were released into the SBF area (Table 2). This resulted in significantly fewer released individuals in SBF in 2015–2016.

In summer 2018, a monitoring approach was used that allowed us to identify the age class of the rabbit (adult or juvenile) based on the pellet size. In the summer monitoring, we identified 2 wild-born rabbits from the winter 2017–2018 monitoring season, 49 new wild-born adult rabbits that were not identified during winter 2017–2018 surveys, and three wild-born juveniles. Most of the rabbits identified in the winter 2018–2019 surveys were new wild-born rabbits (123 rabbits), but 14 of the 15 recaptured individuals (93%) were from the summer 2018 monitoring. The winter 2019–2020 survey indicated a significant decline in the population, with the minimum count of rabbits at eight individuals. Most of the individuals detected were new wild-born rabbits (63%), whereas the other rabbits (38%) were detected in the previous survey year or during the summer 2018 monitoring (Table 2). Averaged across all monitoring years, the sex ratio of all detected rabbits maintained an approximate 1:1 relationship (49.5% males and 50.5% females). However, the male-to-female (M/F) ratio varied by year, where the number of males detected in the surveys initially was lower compared to females during 2012–2015 (range V-1:1.6), but with no significant differences in sex ratio from year to year (*p* = 0.76–1.00; Table 4). The number of males significantly decreased in the summer 2018 survey (1:1.8) from winter 2017–2018 (*p* = 0.007) but returned to male-dominant by winter 2018–2019, producing the largest M/F sex ratio difference (1.9:1).

### 3.2. Genetic Diversity and CB Ancestry at Sagebrush Flat

Genetic diversity across the SBF population has remained relatively consistent across the years for H_o_ and H_e_ ($\bar{H_{o}}=0.74\pm 0.07$, range 0.62–0.84, and $\bar{H_{e}}=0.79\pm 0.03$, range 0.72–0.82) (Table 4), but there was a significant increase in 2017 (from the previous year) to 0.84 (*p* < 0.001). During the summer 2018 surveys, we saw a significant decline in H_o_ to 0.76 (*p* = 0.02) compared to winter 2017–2018 (Table 4). The samples that were collected during this survey effort included adults and juveniles that were closely related, likely causing the decrease in the H_o_. By winter 2018–2019, the H_o_ decreased (*p* < 0.001) compared to winter 2017–2018 to its lowest (0.62) and remained consistently low into the winter 2019–2020 survey period (Table 4). Although there was variability from year to year in H_o_, the decrease in H_o_ over time (2012 compared to 2019) was only marginally significant (*p* = 0.05). AR (5.10 ± 0.21) varied minimally throughout the survey periods from 2012 to 2020, ranging from 4.67 to 5.35 with no significant differences from year to year (*p* = 0.16–0.96).

We documented a decrease in average CB ancestry for the reintroduced population over time that was influenced, in part, by translocations of individuals from other populations outside of Washington. CB ancestry varied from 2012 to 2019, averaging 18.9 ± 10.9% (Table 4). From 2012 to 2016, there were no significant differences in CB ancestry (*p*-values > 0.05). In winter 2017–2018, CB ancestry declined significantly from the previous year (*p* = 0.01) in the identified individuals, resulting in averaged CB estimates of 15.3%. CB ancestry increased significantly (*p* = 0.01) by summer 2018 (18.5%) and was maintained at this higher level each subsequent year. In 2012, only 48.9% of individuals detected in winter surveys had estimates of CB ancestry ≥5% because many of the individuals released were obtained from populations in other states and placed in the onsite breeding enclosures. By 2013, there was an increase to 88.6% of individuals with detectable CB ancestry, but then a decline in 2014 to 71.4%. During 2015–2020, all individuals detected in winter and summer monitoring surveys contained ≥5% CB ancestry. All individuals that were wild-born from 2012 to 2020 contained detectable CB ancestry, except for two individuals in 2014 (Table 4). During 2012–2019, the predominant ancestry in identified rabbits was from the Nevada/Oregon/Idaho group (61.04 ± 14.93%), followed by the Wyoming/N. Utah ancestry was also represented (18.79 ± 13.00%). The S. Utah ancestry had nearly been lost from the SBF population during 2012–2019 (1.98 ± 7.18%). Initially, in 2012, S. Utah ancestry estimates averaged 10.05% but from 2013 to 2019, estimates ranged from 0.33–3.63%.

Effective population size (N_e_) increased from 2012 to 2014, ranging from 15.4–30.4 (Table 4). In winter 2015–2016, N_e_ decreased to 19.3, and the minimum count of rabbits that year was also at its second lowest (n = 18). The lower and upper bounds of the 95% confidence interval fell within or were near the confidence intervals for the previous years. *N*_e_ of the SBF population appeared to peak and stay somewhat consistent in 2016 and 2017, with values ranging from 40.7 to 44.3 individuals and overlapping confidence intervals (2016: 35.0–47.9, and 2017: 40.6–48.5). A decline in the overall *N*_e_ was observed in 2018 (both summer and winter survey estimates staying consistent between 26.9 and 27.6 individuals), and then declined even further in 2019 to 12.3 individuals.

### 3.3. Genetic Monitoring at Beezley Hills

The first attempt to re-establish the BH population occurred during summer 2015 (Table 3), but immediately after the release of rabbits, surveys identified numerous pygmy rabbit carcasses, and it was later determined that nearly all the rabbits released contained lethal to sub-lethal levels of the parasite coccidia (*Eimeria brachylagia*). Additionally, these sick rabbits were released during a drought year [71]. The following winter (2015–2016), informal transect and helicopter surveys were performed, but no rabbits or active burrows were identified (Table 3). In winter 2017–2018, a small survey effort (0.21 km^2^) was conducted because rabbits that were stocked into the new mobile breeding enclosure at BH had escaped. Five escaped individuals were identified during this survey period (Table 3 and Table 5), but there was no evidence of individuals (or their descendants) from earlier releases (2015).

Formal re-establishment of the BH recovery area was attempted again in summer 2018 with the release of 17 individuals. Winter surveys were conducted around the release pens in 2018–2019 (Table 3, Figure S1). Species identification success rates ranged from 80 to 93% ($\bar{X}=86\;\pm 7\%$) with very few cottontail pellets collected (0–2 samples per survey period). Individual identification success rates varied from 88 to 92% ($\bar{X}=90\;\pm 11\%$).

Three individuals were detected in winter 2018–2019 in BH. Two of the individuals were captively bred and released in 2018, whereas the other rabbit was a wild juvenile translocated from the SBF population in 2018 (Table 3 and Table 5). During the winter 2019–2020 surveys in BH, five individuals were identified; four were released enclosure-born rabbits from summer 2019, and the other was a wild juvenile translocated from the SBF population in summer 2019 (Table 3 and Table 5).

Summer monitoring was conducted in BH in 2019, and seven individuals were identified; two (29%) were enclosure-born juveniles that were released that summer, three juveniles (43%) were from the mobile breeding enclosure at BH but had escaped, and two wild-born rabbits (29%) were identified. The wild-born rabbits were determined to be produced by one female and one male rabbit.

### 3.4. Genetic Diversity and CB Ancestry at Beezley Hills

Genetic diversity across the BH population varied from 0.59 to 0.80 across the years, for H_o_ ($\bar{H_{o}}=0.73\pm 0.11$; Table 5). During 2017–summer 2019, H_o_ ranged from 0.75 to 0.80, with no significant differences (*p* > 0.05) until a decline in winter 2019–2020 (0.59) from summer 2019 (0.80; *p* = 0.002). The decrease was not significantly different from diversity in the previous winter (*p* = 0.07) but was different from the initial levels of heterozygosity identified in winter 2017–2018 (*p* = 0.01). AR varied throughout the survey periods during 2017–2019, ranging from 3.71 to 5.41 ($\bar{X}=4.31\;\pm \;0.54$). There was a significant decrease (3.82, *p* = 0.003) in summer 2019, but AR showed no significant difference in winter 2019–2020 (3.71, *p* = 0.73). AR levels during winter 2017–2018 were comparable to SBF values with no significant differences for any given year at SBF (*p* > 0.05). AR values observed during summer 2019 and winter 2019–2020 were significantly lower compared to any year at SBF (*p* < 0.0001).

Due to the small sample size, N_e_ estimates could not be accurately estimated for most years for BH, except for during summer 2019, where N_e_ was estimated to be 9.3 individuals (Table 5). From 2017 to 2019, CB ancestry did not differ significantly among years (*p* = 0.86–0.99) with CB ancestry ranging from 22.87 to 27.46% ($\bar{X}=24.46\;\pm \;7.98\%$) (Table 5). All individuals that have been detected and released into BH retained ≥ 5% of CB ancestry. The predominant ancestry in identified rabbits was Nevada/Oregon/Idaho ($\bar{X}=62.35\;\pm \;9.90\%$, range 40.18–74.75%) and the Wyoming/N. Utah ancestry was still represented across the years ($\bar{X}=10.69\;\pm \;8.08\%$, 1.72–31.84%). The S. Utah ancestry had nearly disappeared from the BH population ($\bar{X}=2.51\;\pm \;1.22\%$, range 1.16–7.00%).

### 3.5. Genetic Monitoring at Chester Butte

The CHB recovery area was established in summer 2018 with the release of 17 juveniles into temporary release pens and then augmented with an additional 21 juveniles in summer 2019 (Table 3 and Table 6). From 2018 to 2020, 1.07–2.43 km^2^ of habitat surrounding the temporary release pens was monitored (Table 3, Figure S2). Species identification success rates ranged from 85 to 97% with very few cottontail pellets collected (0–2 samples per survey period). Individual identity success rates varied from 81 to 84%, and 6–10 individuals were identified (Table 3). All rabbits that were identified during winter surveys were rabbits that had been released into release pens that year. There was no evidence of wild-born rabbits in CHB.

During winter 2018–2019 surveys, six rabbits were identified, of which one individual (16.7%) was an enclosure-born rabbit and the remaining five rabbits (83.3%) were juveniles translocated from SBF. In winter 2018–2019, 10 rabbits were identified either in the release pens or in the wild. Nine of the ten rabbits (90%) were enclosure-born rabbits, and one rabbit (10%) was a juvenile translocated from the wild SBF population.

During summer 2019 surveys, 1.52 km^2^ were surveyed and 20 pellets were collected. Species ID success rates were lower (80%) than winter success rates (avg 96%). A total of 5 individuals were detected, which represented 5 of the 21 (23.8%) juveniles that had been released into pens. No wild-born rabbits were detected.

### 3.6. Genetic Diversity and CB Ancestry at Chester Butte

Genetic diversity across the CHB population has remained somewhat consistent across the years for H_o_ ($\bar{H_{o}}=0.75\pm 0.02$, range 0.73–0.77) (Table 5) with no significant differences detected from year to year or from initial establishment (2018) to winter 2019–2020 (*p* = 0.43–0.93). Mean AR at CHB (4.31) was identical to the mean AR of BH (4.31). AR values ranged from 3.71 to 4.65 from 2018 to 2020, with no significant differences detected (*p* = 0.87), until a decline in winter 2019–2020 (3.69, *p* = 0.02). AR values in CHB were similar to AR in SBF, except for winter 2017–2018 (*p* = 0.04). Winter 2019–2020 values for CHB were significantly lower than all years at SBF (*p* > 0.05). Due to the small sample size, *N*_e_ estimates could not be accurately estimated. From winter 2018–2019 to summer 2019, there was a significant change in CB ancestry (*p* = 0.01), with CB ancestry increasing from 14.85% (winter 2018–2019) to 20.89% (summer 2019) with no significant differences detected in subsequent surveys (Table 5). All individuals that have been detected and released into CHB have contained ≥5% CB ancestry, averaging 18.46 ± 3.45%. The predominant ancestry in identified rabbits is Nevada/Oregon/Idaho ($\bar{X}=67.77\;\pm \;6.41\%$, range 55.42–77.36%) and the Wyoming/N. Utah ancestry is still represented across the years ($\bar{X}=12.05\;\pm \;7.96\%$, ranging from 4.84–27.10%). The S. Utah ancestry has nearly been lost from the CHB population. S. Utah estimates are below the 5% threshold considered to be significant ($\bar{X}=1.73\;\pm \;0.80\%$, range 0.85–3.81%).

### 3.7. Apparent Survival

For rabbits released from 2012 to 2016 at SBF, the apparent survival of released rabbits to the following winter ranged from 0.1% to 39% ($\bar{X}=14\;\pm \;15\%$; Table 2). A total of 141 juveniles were detected from the 1354 juveniles that were released into the SBF area, resulting in an average juvenile apparent survival rate across all winter surveys of 10.4%. As for adults, 125 were released into the SBF area and 25 were detected, resulting in an averaged adult apparent survival rate of 20% across all years. Of the 141 released juveniles that survived their first winter, only 5 released juveniles were detected a second winter, resulting in an average adult apparent survival rate to their second winter of 3.5%; no released adults were ever detected a second winter. As for wild-born rabbits, 420 wild-born individuals have been identified from winter monitoring surveys across the years (Table 2). A total of 20 of the 420 wild-born individuals were detected a second winter after their first detection, resulting in an average adult apparent survival rate to their second winter of 4.8%, although the difference is not significant compared to released juveniles (Fisher’s exact test, *p*-value = 0.64). One juvenile that was detected during the summer 2018 survey was also detected during the winter 2019–2020 survey, 1.5 years later.

The year in which rabbits were released played a significant role in apparent survival for juvenile rabbits in SBF (Table S1). During 2012–2014, there was a positive influence of year on apparent survival; 2015 had the largest negative effect on apparent survival, but 2016 also reduced apparent survival, although estimates for 2016 overlapped zero. Released juvenile survival was positively influenced by release day and release weight and negatively influenced by homozygosity (Figure 3, Tables S1 and S2). Weight and release day were moderately correlated (Pearson’s correlation coefficient = 0.62, *p* < 0.0001). Rabbits that were released after the breeding season ended in July weighed more, driving this correlation. Sex and CB ancestry appeared in the top model sets, but their addition to the top model did not improve the log-likelihood and 95% confidence intervals around the model-average estimates (Table 6). These parameter estimates overlapped zero, suggesting that they are not actually significant in the model [72]. Apparent survival of released adults in the winter following their release was influenced by release day only (Figure 3, Table S2 and Table 6). Of the 44 individuals that were released earlier in the year in the larger data set (2014), only 7% were detected in winter; whereas the 45 adults released later in the year had a 47% detection rate. Genetic diversity showed weak evidence for a positive effect on adult survival (Table S3 and Table 6) but still overlapped zero for its parameter estimates.

## 4. Discussion

Our study intensively and effectively applied genetic tools to monitor demographic and genetic parameters of the Columbia Basin pygmy rabbit recovery program for eight years following reintroductions. Monitoring methods were designed in collaboration with managers, and frequent updates of results were provided to allow adaptive management of this endangered population. Noninvasive genetic sampling has allowed us to monitor the wild populations for spatial expansion, apparent survival of released individuals, genetic diversity and ancestry, minimum population size, effective population size, and reproduction in the wild, a critical parameter for success. We monitored the spatial distribution of rabbits across SBF, identifying a striking shift in the use of habitat in 2015 from state-protected mature sagebrush habitat to CRP fields that had been planted with sagebrush and forbs 20–30 years previous, providing insight into habitat preference for conservation actions. By monitoring individual rabbits, we documented reproduction in the wild and determined that survival to a second detection year did not significantly differ between wild-born rabbits and released rabbits. We documented the persistence of CB ancestry in wild populations over the eight years since the first reintroduction, and by 2015, all individuals detected in winter survey efforts contained detectable CB ancestry (>5%).

Through our genetic monitoring, we modeled apparent survival in SBF and determined that release day, release weight, and genetic diversity positively influence apparent survival in released juvenile rabbits, and only release day positively influences apparent survival in released adults. The significant negative relationship between apparent survival and individual homozygosity indicates the presence of inbreeding depression and demonstrates the value of maintaining genetic diversity in this population. Our results can inform the design of future releases to possibly increase the survivorship of released rabbits. Additionally, we were able to estimate an average number of rabbits to the number of active burrows found in each survey region, allowing managers to determine an approximate number of rabbits in each area from field surveys, if genetic monitoring efforts cannot be conducted. Using genetic sampling of both tissue and fecal pellets, we have effectively and efficiently monitored the endangered Columbia Basin populations, providing critical information for adaptive management of this reintroduced species.

### 4.1. Habitat Use and Population Density

Contrary to the results of other studies [73], our data shows more active burrows in disturbed habitats (defined as any non-shrub-steppe/CRP habitat within a seasonal home range radius around the active burrow) than intact native shrub-steppe habitats found within SBF. As hypothesized, active burrows within the SBF population were predominantly detected within the SBF native shrub-steppe habitat from 2012 to 2014, close to release sites, with minimal detections in CRP. Yet, as the population began to increase, habitat use shifted spatially to CRP starting in 2015. As wild-born rabbits continued to expand their distribution and recolonize habitat, this shift to CRP habitat with early successional stages of replanted sagebrush raises many questions as to the reasons behind the move. WDFW considers CRP habitat to be highly fragmented and patchy, since patch sizes are small, and most areas are surrounded by agricultural fields [74]. Most literature suggests fragmentation negatively affects specialist species, including pygmy rabbits [73].

However, many sagebrush steppe species, including sage grouse (*Centrocercus urophasianus*; [75]), mule deer (*Odocoileus hemionus*), and jackrabbits [76] have chosen to occupy CRP habitat, containing early successional sagebrush, over adjacent, undisturbed, mature sagebrush habitat. Additionally, increased nest survival has been documented in CRP habitat in Brewer’s sparrows (*Spizella breweri*) and sage thrashers [77]. CRP fields may help connect fragmented patches of shrub-steppe habitat, creating a relatively continuous vegetative community for the dispersal of sagebrush obligates (i.e., [78]). Also, preliminary data of terrestrial predator visitations at pygmy rabbit burrows in 2017–2018, using game cameras, revealed significantly fewer terrestrial predator occurrences near CRP burrows compared to SBF burrows in summer and fall months, but by winter, predator occurrences at burrow sites were similar between SBF and CRP [74].

During our study in Washington, pygmy rabbits were identified at 1–6 burrow systems within a winter survey period; this finding is comparable to the number of burrow systems used by rabbits within their home range during non-breeding seasons in Idaho pygmy rabbits [79]. Pygmy rabbits are typically not observed together at burrow systems and are known to occupy more than one burrow system, swapping throughout the year [79,80]. Home ranges during winter months have been shown to be more restricted than other seasons [79,81]. Through our pellet surveys, from 2012 to 2020, the number of rabbits identified per the number of active burrows identified averaged 0.63 rabbits/burrow with a range of 0.33–1.00 across all populations. Further analysis of this information can provide a means for estimating the relative abundance through burrow counts, rather than relying strictly on genetic monitoring. Burrow counts for indexing abundance have been evaluated previously in pygmy rabbits and revealed that the density of burrows can serve as an index for monitoring changes in abundance of pygmy rabbits in eastern Idaho, although their models were based on radio-collared rabbits [82].

SBF/CRP density estimates ranged from 0.01 to 0.09 rabbits/ha, CHB was 0.01 rabbits/ha, and BH ranged from 0.04 to 0.09 rabbits/ha. Our findings are similar to lower density estimates for specific regions in Idaho. Density estimates for seven established populations within east central Idaho ranged from 0.02 to 0.46 rabbits/hectare [82]. CHB estimates are low compared to the Idaho, SBF/CRP, and BH estimates, but this population is only in its first two years of establishment. The CHB habitat has the greatest potential for expansion and population growth of pygmy rabbits due to the continuous sagebrush-steppe habitat in the area [74]. BH, on the other hand, may have higher density estimates due to the much smaller size of the recovery area (79 ha). Potential habitat has been identified in CRP private land parcels surrounding BH, and a large number of active pygmy rabbit burrows have been identified to the east of the BH recovery area.

### 4.2. Survival and Inbreeding Depression

Pygmy rabbits have a low and variable annual survivorship rate, documented at 0.3–17% in Nevada/Oregon [83] and 7–45% in Idaho [81]. Juvenile mortality in Idaho was 69% and 89% for males and females, respectively, with the highest mortality occurring within the first two months of emergence from natal burrows [84]. Within the SBF wild population, the average survival rate of identified individuals during winter monitoring surveys was 14% from 2012 to 2016. Each year that rabbits were released, we detected a decreasing trend in survivorship of released individuals. The very low 1% apparent survival rate of released individuals in 2015 may be attributed to a combination of the sub-lethal to lethal levels of coccidia identified in released individuals, a drought year, low individual identification success rates due to unfavorable weather conditions, and lower survey efforts compared to 2014 [56,71]. The decreasing trend of survivorship among enclosure-born, released rabbits continued with second year detection, although the 4% second year detection was comparable to wild-born rabbits (5%). The release year for juvenile rabbits in SBF was highly significant and was retained in each of the apparent survival models, suggesting that other environmental variables across that landscape in a given year play a role in the apparent survival of released rabbits. The decrease in survival after the first year could be explained by an increased response of predators across the reintroduction landscape, as has been documented in other studies [48,85,86,87]. Differences in predator densities may have also led to the shift in spatial distribution across the landscape in 2015.

Timing of the release date for both adults and juveniles significantly influenced apparent survival at the SBF population and in the release pens at CHB and BH. The later they were released, the greater their chances of being detected in winter survey efforts, likely due to the decreased intervals of being exposed to predation, especially raptors [83,88] and other mortality sources. High mortality rates typically occur in juveniles during the two months following emergence from natal burrows [84]. Thus, allowing rabbits to develop longer in the breeding enclosures or temporary release pens may increase their overall survivorship to winter. Multiple factors might influence variation in survival of leporids spatially and temporally, including variability in predator populations, climatic conditions, forage quality or quantity, soil characteristics, parasites, and disease [51,89,90,91]. Juvenile apparent survival was also positively influenced by release weight and release age; the older the rabbit, the more it weighs. DeMay et al. [48] raised the concern that juveniles kept in enclosures longer may have lower survival due to possible acclimatization to humans and decreased exposure to predators, but as their model and ours showed, there appears to be no acclimatization effect. By keeping juveniles in the enclosures longer, juvenile body condition and weight could increase, increasing their overall chances of survival.

Increasing homozygosity significantly decreased the apparent survival rate of juveniles in the SBF population. Although not significant, homozygosity followed the top model in adults as well, suggesting overall genetic diversity may play a role in survivorship of all life stages. Increased individual heterozygosity has also been shown to be an important indicator of survival in the translocation of Mojave Desert tortoises (*Gopherus agassizii*) [50]. The influences of increased genetic diversity may be attributed to preventing the effects of inbreeding in the population, favoring those of higher genetic diversity from random mating [92], and thus preventing a deleterious effect on population fitness [93]. Low levels of genetic diversity and high levels of inbreeding are thought to have contributed to the low reproductive success and juvenile survival in the pygmy rabbit captive breeding program [45,94]. The genetic rescue using Idaho rabbits conducted in 2001–2002 helped increase genetic diversity, increasing pregnancy rates and juvenile growth and survivorship within the captive breeding program [45].

### 4.3. Prospects for Population Persistence

During 2012–2014, the number of wild-born rabbits identified was only 14, and as DeMay et al. [48] suggested, the SBF population did not appear to be sustainable due to the low apparent survival and reproduction rates. In 2015, the wild-born rabbit total (n = 16) surpassed the 2012–2014 total and continued to increase during 2015–2018, with 401 wild-born rabbits identified. This suggests that the SBF population may be in the early stages of being a sustainable wild population since reproduction rates have significantly increased since the findings in DeMay et al. [48], although apparent survival rates have not changed (13% to 14%). Additionally, due to the small population size at SBF, the population is vulnerable to other stochastic effects, as we saw in the major decline in winter 2019–2020, and as fires have negatively impacted reintroduction efforts in both BH and CHB. Most rabbits that are found each year during survey efforts appear to be new wild-born juveniles, rather than adults that have survived multiple years. Annual survival rates of radio-collared pygmy rabbits ranged from 7 to 45% in Idaho [81] and from 0.3 to 17% in Oregon and Nevada [83]. Although we are using pellets to assess apparent survival and thus likely underestimate survival, our estimates fall in these ranges, demonstrating the value of this noninvasive genetic sampling approach.

As a result of the increased reproductive productivity observed in the SBF wild population and declining numbers in the breeding enclosures, supplemental releases were halted in 2017. Unfortunately, a significant decrease in numbers of rabbits (~94% decline in individuals detected) was detected in winter 2019–2020. The causes of this decline are unknown. One possibility is that heavy flooding in March 2019 from the large amount of snow received in February negatively affected juvenile rabbits in the natal burrows. A total of 26% of released adults and 38% of released juveniles dispersed ≥1 km, suggesting that some rabbits have dispersed beyond the SBF/CRP survey areas [95]. Further efforts, such as helicopter surveys, drone surveys, and the use of conservation canine units, could increase the efficiency and spatial extent of the search for active burrows. In fact, surveys from 2021 to 2025 have found them in 8 habitat patches that have been recolonized naturally. These patches range from 1.5 to 10.2 km and average of 4 km dispersal to other habitat patches.

At the end of our study, BH and CHB populations were still in early stages of establishment and could not yet be considered sustainable wild populations. Pygmy rabbits had not resided in CHB since the 1980s, and most of the burrows that were identified during surveys were either newly created or modified badger digs. By 2019, no wild-born individuals were detected in CHB, and only two wild-born rabbits were detected in BH. In the SBF population, wild-born rabbit production did not significantly increase until its fifth year; thus, we can expect a similar pattern in these reintroduction areas. Initial attempts in 2015 to establish a population in BH were unsuccessful due to translocated juveniles being infected with coccidia [56]. Both areas were early in their establishment (≤5 years) and follow similar trends from the SBF population, in which the majority of rabbits detected were ones released that same year (Table 2). Summer monitoring of both of these populations was completed in 2020 and will provide insight into how rabbits are spatially distributing across the habitat and if wild reproduction is beginning to increase. Unfortunately for the CHB population, in September 2020, the Pearl Hill fire swept through the CHB area, destroying nearly 97,124 ha [96]. All wild rabbits, release pen rabbits, mobile enclosures, and the larger enclosure were destroyed. CHB had the greatest overall potential for expansion due to the large amount of connected sagebrush steppe habitat in the state of Washington. This loss was a great hit to the Columbia Basin pygmy rabbit recovery program.

### 4.4. CB Ancestry and Genetic Diversity

The CB pygmy rabbit population has undergone both genetic (2001) and demographic rescues (2011), contributing to the increase of genetic diversity compared to that observed in individuals sampled in CB population prior to reintroduction [39]. Previous work [55,63] detected four distinct genetic ancestries in our mixed ancestry rabbits: (1) CB, (2) Nevada, Oregon, and Idaho, (3) northern Utah and Wyoming, and (4) southern Utah. Although CB ancestry played a role in the second top model in juvenile apparent survival in SBF, it did not significantly influence apparent survival of adults or juveniles in any of the models. DeMay et al. [55] provided evidence of fitness benefit associated with Columbia Basin ancestry in the enclosure populations. Males with Columbia Basin ancestry estimates had increased reproductive output, whereas males with high levels of northern Utah/Wyoming ancestry and females with high levels of Nevada/Oregon ancestry had decreased levels of reproductive output. Within the SBF population, all wild-born rabbits detected, other than two individuals in winter 2014-15, contained CB ancestry greater than 5%. This supports our hypothesis that CB ancestry would be maintained in this reintroduced population, despite the overall decrease in percentage representation per rabbit. Since 2015, all individuals detected during winter or summer monitoring surveys contained CB ancestry, suggesting that selection may be favoring ancestry in the wild population. Managers must balance the needs for demographic rescue and the numbers of reintroduced individuals with the preservation of locally adapted genes. Introducing genetically divergent or geographically distant individuals into a population can cause outbreeding depression, a decrease in fitness caused by the breaking up of co-adapted traits or the loss of locally adapted alleles [27,97,98].

However, overall estimates of heterozygosity and AR did not significantly differ across the 8 years within SBF/CRP, providing evidence that genetic diversity has been maintained within the wild population in contrast to our hypothesis that it would decline. AR values within the SBF/CRP population reflected the AR values from the breeding enclosures, since the population was founded and augmented with individuals from each enclosure. Heterozygosity levels for both the wild and enclosure populations have nearly doubled (H_o_ = 0.62–0.84) compared to the estimates from the remnant SBF population in 2001 H_o_ = 0.40; [40]. Reintroduction efforts are often challenged by a small number of founders and the rapid loss of genetic diversity [36,99,100,101]. Additionally, reintroductions of pygmy rabbits are often accompanied by high mortality rates during the rabbit’s first year [48,55,83,84], which may reduce the effective population size and genetic diversity within the wild population.

The BH and CHB populations had many fewer founders compared to the SBF population, resulting in the lower AR (4.31 alleles per locus), yet observed heterozygosity (H_o_ = 0.74) was comparable to SBF/CRP. AR and heterozygosity were comparable to estimates found in Idaho (H_e_ = 0.73 across all sites, allelic richness = 4.3–5.6; [102]), but much higher compared to the Wyoming populations (H_e_ = 0.58, AR = 2.8–3.1; [103]). However, results are not directly comparable because both studies used a subset of our loci (n = 10). The genetic and demographic rescues performed in 2001 and 2011, respectively, successfully increased and maintained the genetic diversity within the captive and wild CB populations.

In support of our hypothesis, all *N_e_* point estimates for the SBF/CRP populations were under 50 individuals, and all upper estimates in the 95% confidence intervals were still under 50. For many species, an effective population size greater than *N_e_* > 100 is considered sufficient for short-term persistence of a population, preventing inbreeding depression [104], yet in highly dynamic populations, *N_e_* > 300 is recommended [105,106]. Yearly N_e_ estimates within SBF are similar to those found in the small and endangered (state-listed) populations of New England cottontail (average *N_e_* = 3.2–36.7; [106,107]). Concern about the persistence of all CB pygmy rabbit populations should be a major priority, and augmentation into each of the populations may be necessary to maintain genetic diversity for the unforeseeable future, until N_e_ estimates increase.

### 4.5. Management and Conservation Implications

The use of genetic monitoring has greatly increased in conservation biology and wildlife management [3,4] and is becoming widely used in monitoring and adaptive management of reintroduced populations [108,109,110,111,112,113,114]. Our study has helped effectively guide adaptive management strategies for the Columbia Basin pygmy rabbit recovery program. Characteristics of the pygmy rabbit reintroduction that helped retain high genetic diversity included a large founding population from multiple sources, supplementation of more animals into the wild each year, short generation times, promiscuous mating systems [48,49], and high reproductive output. Additionally, a portion of juveniles known to have high Columbia Basin ancestry were retained in the breeding enclosures each year for future breeding in relatively safe conditions compared to the wild, thereby retaining more Columbia Basin ancestry for future releases.

Evaluating the genetic diversity present in both the founding population and subsequent generations of the reintroduced populations allowed us to monitor the population’s genetic response to reintroduction and assess the success of the reintroduction in genetic and demographic terms. One of the main goals of the Columbia Basin pygmy rabbit recovery program was to maintain the Columbia Basin ancestry, and our monitoring data has shown that nearly all wild-born rabbits (99.3%) have maintained >5% native CB ancestry. We acknowledge that ancestry estimates based on 18 microsatellite loci can be imprecise and have wide confidence intervals; thus, we are currently using reduced representation sequencing approaches [115] to identify thousands of single-nucleotide polymorphism loci (SNPs) from the founders of this population that can be used for future ancestry estimates. Also, further investigation of adaptive loci is necessary to understand which regions of the genome are under selection within the Columbia Basin population and how the genetic diversity of these lo56ci has responded to the reintroduction effort.

The SBF population showed initial signs of being self-sustaining based on high reproductive rates, moderate survival rates, and large numbers of wild-born rabbits identified from 2015 to 2019. However, based on the population crash in 2019 and *N_e_* estimates, augmentation of the population may be needed in the future. Noninvasive genetic sampling has proven to be an effective and efficient tool in monitoring this reintroduced population and in helping managers address the goal of the Columbia Basin recovery project of establishing multiple sustainable wild populations within the sagebrush steppe habitat of Washington. The results of this study have helped effectively guide monitoring strategies in the past and can be used to inform future recovery efforts for the CB pygmy rabbit. This study can also serve as a model for other genetic monitoring and management studies for reintroduced populations.

## Figures and Tables

**Table 1:** Study objectives and parameters examined for endangered Columbia Basin pygmy rabbits (*B. idahoensis*) of Washington, and the genetic monitoring approaches used to address each parameter. Wild population is defined as all free-ranging rabbits on the Sagebrush Flat wildlife area in central Washington.

Objective	Parameter	Genetic Monitoring Approach
***Wild*** ***Apparent*** ***Survival***	Released Individuals	Compare tissue sample genotypes to winter pellet genotypes
	Adults After 1st Winter	Compare tissue sample genotypes to winter pellet genotypes
	Factors Influencing Survival Rates (Genetic)	Logistic regression models of winter monitoring data
***Wild*** ***Population*** ***Information***	Habitat Occupancy and Spatial Distribution	Compare GPS locations of burrows and identified species and individuals from winter monitoring data each year
	Minimum Population Size	Winter monitoring fecal DNA genotyping
	Sex Ratios in Wild Population	Ratio of male/female individuals identified in winter monitoring surveys
	Rabbits Per Active Burrow	Ratio of number of rabbits identified to total number of active burrows located during winter monitoring surveys
	Genetic Diversity	Estimates of observed and expected heterozygosity, and allelic richness from winter and summer fecal DNA genotypes
	CB Ancestry	Genetic estimates based on winter and summer monitoring fecal DNA genotypes
	Effective Population Size	Parametric point estimates using linkage disequilibrium method and minor allele frequency 0.05, from winter and summer monitoring fecal DNA genotypes

**Table 2:** Details for winter and summer monitoring of pygmy rabbits (*B. idahoensis*) from 2012 to 2020 for Sagebrush Flat/Conservation Reserve Program (CRP) areas in central Washington, USA. Area surveyed (AS) represents ground survey efforts. Release period indicates when juveniles and adults were released into the SBF area and number of adults (AD) and juveniles (JV) released (Released). ENC represents the of enclosures that were in production for the breeding season. Total fecal samples (Fecal Samples) represent all pellet samples collected, and pygmy fecal samples (Pygmy Fecal) represent pellets that were determined to be pygmy rabbit through species identification (SPID) or microsatellite panels. SPID success rates (SPID Success) were not formally introduced until winter 2018–2019 survey year. Individual identification success rates (ID Success) are based on the number of individuals identified from confirmed pygmy rabbits. Unknown parentage represents individuals whose parents could not be assigned at the 95% confidence interval. Individuals detected represent the number of individuals that were identified through fecal pellets and the year represented in () represents the year in which the identified pygmy rabbit was either released or first detected as a wild-born individual. The contributing breeders of wild-born juveniles represents the number of individuals (parents) that were detected through parentage assignments of all new wild-born individuals detected.

Breeding Season	ENC	Release Period	Released	Survey Period	AS	Fecal Samples	Pygmy Fecal	SPID Success	ID Success	Individuals Detected (Year Released or First Detected)	Contributing Breeders of Wild-Born Juveniles
**2012**	2	May–July	104 JV	Dec 2012– Jan 2013	9.71 km^2^	117	111	NA	78%	45	1 female
			0 AD							41 released (2012)	1 male
										4 wild-born (2012)	
**2013**	3	May–August	265 JV	Jan–Feb 2014	10.52 km^2^	296	273	NA	46%	44	7 females
			7 AD							3 released (2012)	7 males
										34 released (2013)	
										7 wild-born (2013)	
**2014**	4	March– November	717 JV	Jan–Mar 2015	13.76 km^2^	265	212	NA	76%	91	2 females
			113 AD							1 released (2013)	3 males
										87 released (2014)	
										3 wild-born (2014)	
**2015**	4	February–October	149 JV	Jan–Feb 2016	10.84 km^2^	105	105	NA	20%	18	11 females (1 unknown)
			4 AD							1 released (2014)	8 males (5 unknown)
										1 released (2015)	
										16 wild-born (2015)	
**2016**	4	May–October	119 JV	Dec 2016– Mar 2017	24.28 km^2^	193	124	46%	52%	60	18 females (25 unknown)
			1 AD							1 wild-born (2015)	17 males (32 unknown)
										5 released (2016)	
										54 wild-born (2016)	
**2017**	4	May–October	0 JV	Dec 2017– Mar 2018	14.67 km^2^	357	296	72%	56%	158	47 females (98 unknowns)
			0 AD							2 wild-born (2016)	46 fathers (92 unknowns)
										156 wild-born (2017)	
**2018**	2	May–August	0 JV	June–Aug 2018	7.40 km^2^	98	98	NA	56%	54	19 females (33 unknowns)
			0 AD							2 wild-born (2017)	
										49 wild-born adults (2017)	17 males (31 unknowns)
										3 wild-born juveniles (2018)	
				Dec 2018– Apr 2019	11.51 km^2^	447	296	77%	73%	138	19 females (88 unknowns)
										1 wild-born (2016)	
										14 wild-born (2017)	20 males (81 unknowns)
										123 wild-born (2018)	
**2019**	2	May–August	0 JV	Jan–Mar 2020	5.89 km^2^	59	27	97%	83%	8	2 females (3 unknowns)
			0 AD							3 wild-born (2018)	
										5 wild-born (2019)	3 males (2 unknowns)

**Table 3:** Winter and summer monitoring of pygmy rabbits (*B. idahoensis*) information from 2012 to 2020 for Beezley Hills (BH) and Chester Butte (CHB) recovery areas. Area surveyed (AS) represents formal survey efforts and helicopter surveys. Release period indicated when juveniles and adults were released into the release areas (either BH or CHB) and survey period represents the timeframe in which monitoring occurred for each year. Released individuals is the number of pygmy rabbits that were either enclosure born (ENC) (adults (AD) or juveniles (JV)) or translocated wild pygmy rabbits captures (WLD) into the release area. Fecal samples represent the total number pellet samples collected and pygmy fecal represents pellets that were determined to be pygmy rabbit through species identification (SPID) or microsatellite panels. SPID success rates (SPID Success) were not formally introduced until winter 2018–2019 survey year. Individual identification success rates (ID Success) are based on the number of individuals identified from confirmed pygmy rabbit samples. The individual detected represents the number of individuals that were identified through fecal pellets and the year represented in () is the year in which the identified pygmy rabbit was either released, as an enclosure (ENC) rabbit or translocated wild captured rabbit (WLD), or year the pygmy rabbit was first detected as a new wild-born individual. Unknown parentage represents individuals whose parents could not be assigned at the 95% confidence interval. * Summer and winter monitoring efforts occurred in 2019. The number of released individuals into each release area is provided in the summer survey period information. The of contributing breeders of wild-born juveniles represents the number of individuals (parents) that were detected through parentage assignments of all new wild-born individuals detected.

Breeding Season	Release Area	Release Period	Released	Survey Period	AS	Fecal Samples	Pygmy Fecal	SPID Success	ID Success	Individuals Detected (Year Released)	Contributing Breeders to Wild-Born Juveniles
**2015**	BH	February–May	369 JV	Jan–Feb 2016	3.09 km^2^	0	0	-	-	-	-
			51 AD								
**2017**	BH	May–October	37 JV	Dec 2017–Mar 2018	0.21 km^2^	9	8	-	75%	5	0 females
			0 AD							5 released ENC (2017)	0 males
**2018**	BH	May–August	14 JV	Dec 2018–Apr 2019	0.69 km^2^	10	8	80%	88%	3	0 females
			11 ENC							2 released ENC (2018)	0 males
			3 WLD							1 released WLD (2018)	
	CHB	May–August	17 JV	Dec 2018–Apr 2019	1.07 km^2^	20	19	95%	84%	6	0 females
			8 ENC							1 released ENC (2018)	0 males
			9 ENC							5 released WLD (2018)	
**2019**	BH	May–August	14 JV	June–Sept 2019	0.69 km^2^	34	27	85%	67%	7	1 female (1 unknown)
			10 ENC							2 released ENC (2019)	1 male (1 unknown)
			4 WLD							3 escaped ENC (2019)	
										2 wild-born (2019)	
	CHB	May–August	20 JV	June–Sept 2019	1.53 km^2^	20	14	80%	93%	5	0 females
			19 ENC							5 released ENC (2019)	0 males
			1 WLD								
	BH	May–August	*	Oct 2019–Feb 2020	0.69 km^2^	15	13	93%	92%	5	0 females
										4 released ENC (2019)	0 males
										1 released WLD (2019)	
	CHB	May–August	*	Oct 2019–Feb 2020	2.43 km^2^	39	37	97%	81%	10	0 females
										9 released ENC (2019)	0 males
										1 released WLD (2019)	

**Table 4:** Winter and summer monitoring of pygmy rabbits (*B. idahoensis*) for Sagebrush Flat/CRP recovery area, in central Washington state, from 2012 to 2019. Minimum count is established through the number of identified pygmy rabbits through genotyping using a microsatellite panel. The number of rabbits per number of active burrows identified is based on the minimum count of rabbits/total number of active burrows located. Density estimates (rabbits/ha) are based on the minimum count/total potential habitat in SBF/CRP (1780 ha). Individuals containing Columbia Basin (CB) ancestry are defined as rabbits with 5–80% CB ancestry using STRUCTURE. Genetic diversity estimates are summarized as observed heterozygosity, expected heterozygosity, and allelic richness. Effective population size is represented at the point estimate and the 95% confidence interval in parentheses using the linkage disequilibrium method and minor allele frequency of 0.05.

		*YEAR*								
		2012	2013	2014	2015	2016	2017	2018		2019
		*SURVEY PERIOD*								
		Winter	Winter	Winter	Winter	Winter	Winter	Summer	Winter	Winter
Category	Parameter									
**Demographic**	Minimum count	45	44	91	18	60	158	54	138	8
	M/F Sex Ratio (actual numbers)	1:1.5 (18:27)	1:1 (22:22)	1:1.1 (44:47)	1:1.6 (7:11)	1.1:1 (32:28)	1.8:1 (101:57)	1:1.8 (19:35)	1.9:1 (91:47)	1:1 (4:4)
	Rabbits/Active Burrow	0.87	0.75	0.63	1	0.92	0.96	0.56	0.65	0.33
	Density (rabbits/ha)	0.03	0.02	0.05	0.01	0.03	0.09	0.03	0.08	0.004
**Genetic**	Average CB ancestry	19.69%	19.72%	19.10%	21.06%	21.89%	15.31%	18.48%	17.97%	16.1%
	% of identified individuals containing CB ancestry	48.89%	88.64%	71.43%	100%	100%	100%	100%%	100%	100%
	% of wild-born individuals containing CB ancestry	100%%	100%	33.33%	100%	100%	100%	100%%	100%	100%
	Effective population size (95% Confidence Interval)	15.4 (13.7–17.3)	29.6 (25.3–34.9)	30.4 (27.7–33.5)	19.3 (14.7–27.0)	40.7 (35.0–47.9)	44.3 (40.6–48.5)	36.9 (23.7–30.8)	27.6 (25.5–29.9)	12.3 (7.0–26.8)
	Observed Heterozygosity	0.76	0.81	0.81	0.75	0.7	0.84	0.76	0.62	0.64
	Expected Heterozygosity	0.80	0.79	0.80	0.80	0.80	0.82	0.79	0.79	0.72
	Allelic Richness	5.22	5.15	5.13	5.29	5.16	5.35	5.00	4.95	4.67

**Table 5:** Winter and summer monitoring of pygmy rabbits (*B. idahoensis*) for Beezley Hills (BH) and Chester Butte (CHB) recovery areas in central Washington state from 2017 to 2019. Minimum count is established through the number of identified pygmy rabbits through genotyping of the microsatellite panel. The number of rabbits per number of active burrows identified is based on the minimum count of rabbits/total number of active burrows located. Density estimates (rabbits/ha) are based on the minimum count/total potential habitat in BH (83 ha) or CHB (893 ha). Individuals containing Columbia Basin (CB) ancestry are defined as rabbits with 10.8–42.74% CB ancestry using STRUCTURE. Genetic diversity estimates are given through observed heterozygosity and allelic richness. Expected heterozygosity is only given when sample sizes are ≥5. Effective population estimates were based on the linkage disequilibrium method and minor allele frequency of 0.05 for minimum counts ≥ 7.

		*SURVEY PERIOD*							
		Winter 2017–18		Winter 2018–19		Summer 2019		Winter 2019–20	
	LOCATION	CHB	BH	CHB	BH	CHB	BH	CHB	BH
***Demographic Parameters***									
Minimum count		-	5	5	3	5	7	10	5
M:F Sex Ratio (actual #s)		-	1:1.5 (2:3)	1.5:1 (3:2)	1:2 (1:2)	1:1.5 (2:3)	6:1 (6:1)	1:1.5 (4:6)	1:1.5 (2:3)
Rabbits/Active Burrow		-	0.42	0.45	0.43	0.5	0.7	0.35	0.5
Density (rabbits/ha)		-	0.06	0.01	0.04	0.01	0.09	0.01	0.04
***Genetic Parameters***									
Average CB ancestry		-	23.98%	14.85%	23.97%	20.89%	22.87%	19.04%	27.46%
% of identified individuals containing CB ancestry		-	100%	100%	100%	100%	100%	100%	100%
Effective population size (95% Confidence Interval)		-	-	-	-	-	9.3 (3.3–32.0)	12.5 (7.1–26.6)	-
Observed Heterozygosity		-	0.78	0.77	0.75	0.73	0.8	0.74	0.59
Expected Heterozygosity		-	0.71	0.71	-	0.66	0.66	0.64	0.64
Allelic Richness		-	5.41	4.65	-	4.59	3.82	3.69	3.71

**Table 6:** Model averaged parameter estimates for each of the parameters describing apparent survival in juvenile and adult released pygmy rabbits (*B. idahoensis*) into the Sagebrush Flat/CRP recovery area (2012–2016) and the apparent survival rates in juvenile rabbits released into pens at the Chester Butte and Beezley Hills recovery areas (2018–2019). Parameter estimates were averaged across all of the candidate models, which were generated by adding weight to the top model according to AICc values. Parameters that overlap zero do not fall into the 95% confidence interval. HL represents homozygosity per locus, an estimate of the genetic diversity, and Columbia Basin ancestry (CB) represents the proportion of ancestry.

Variable	Juvenile Estimate (SBF)	95% CI		Adult Estimate (SBF)	95% CI		Juvenile Estimate (Release Pens)	95% CI	
		Lower	Upper		Lower	Upper		Lower	Upper
Release Day	0.010	0.005	0.014	0.017	0.007	0.027	0.020	−0.005	0.046
Release Weight	0.003	0.001	0.005	0.000	−0.005	0.005	0.000	−0.007	0.007
Sex (female)	0.056	−0.318	0.430	0.109	−0.768	0.986	0.282	−0.786	1.350
HL	−1.905	−3.465	−0.346	−2.442	−6.065	1.180	1.537	−3.588	6.661
CB	0.007	−0.004	0.018	−0.012	−0.039	0.016	−0.038	−0.184	0.109
Year 2012	2.227	1.717	2.737	NA	-	-	NA	-	-
Year 2013	0.544	0.066	1.021	NA	-	-	NA	-	-
Year 2015	−3.982	−5.964	−2.000	NA	-	-	NA	-	-
Year 2016	−0.586	−1.638	0.465	NA	-	-	NA	-	-

## Data Availability

R scripts used for data analysis are available on GitHub (https://github.com/sakura81/PYRA_Genetic_Monitoring, accessed on 6 July 2025).

## Supplementary Materials

The following supporting information can be downloaded at: https://www.mdpi.com/article/10.3390/genes16080956/s1, Figure S1. Pygmy rabbit burrow locations for BH, Figure S2. Pygmy rabbit burrow locations for CHB, Table S1. AIC values for juvenile survival, Table S2. AIC values for adult survival.
