# Supplementary material for: Long-Term Noninvasive Genetic Monitoring Guides Recovery of the Endangered Columbia Basin Pygmy Rabbits (Brachylagus idahoensis)
Source: Genes (Basel). 2025 Aug 13;16(8):956. doi: 10.3390/genes16080956 (PMC12386180; doi:10.3390/genes16080956)
Supplement: Supplementary file 1 [file genes-16-00956-s001.zip › genes-3774149-supplementary.pdf]

Nerkowski et al. Supplemental Material

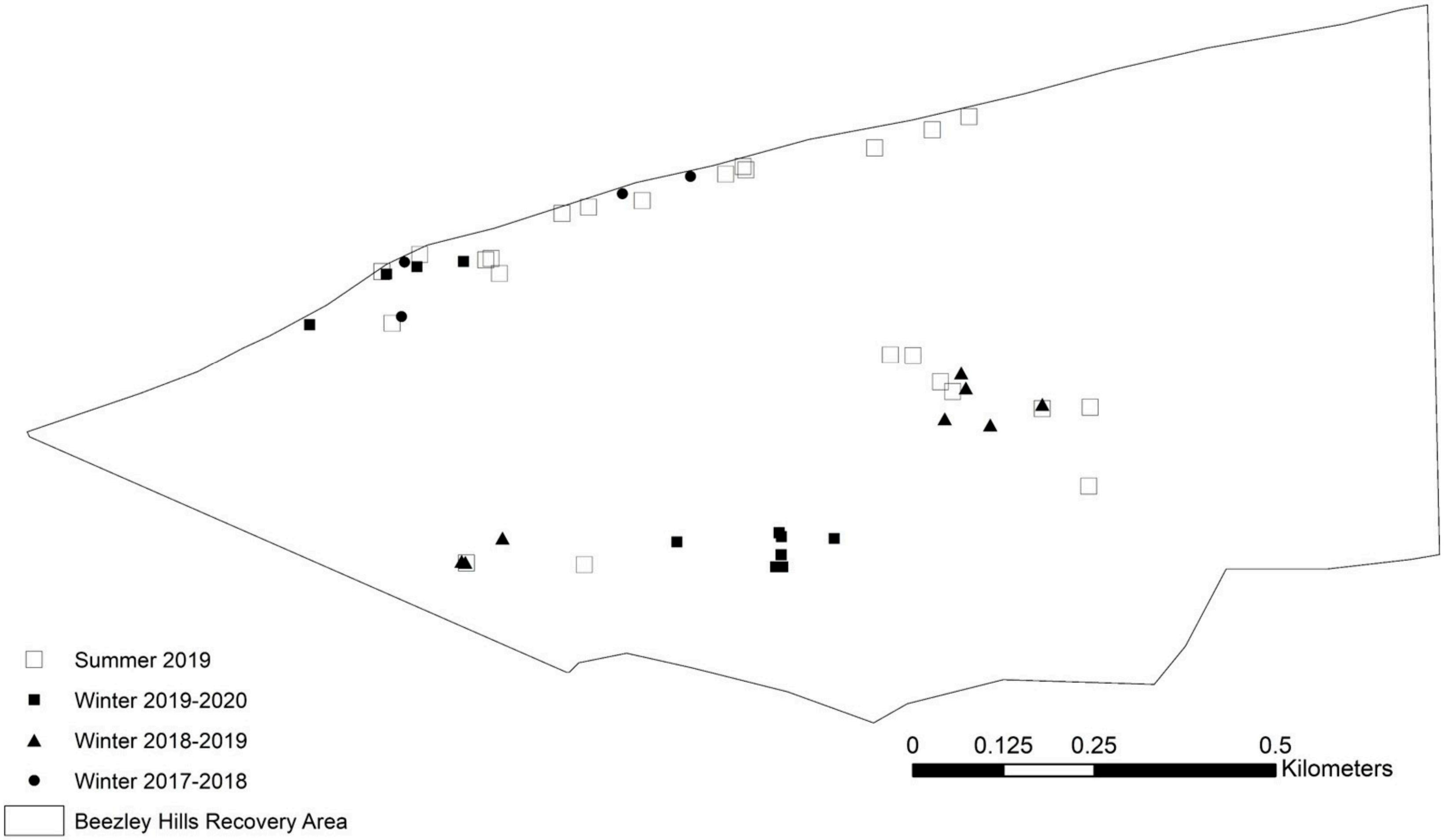

Figure S1. Location of active pygmy rabbit (*Brachylagus idahoensis*) burrows across the habitat identified during monitoring surveys in the Beezley Hills recovery area in central Washington state, during winter 2017-2018 (●), winter 2018-2019 (▲), summer 2019 (■), and winter 2019-2020 (□).

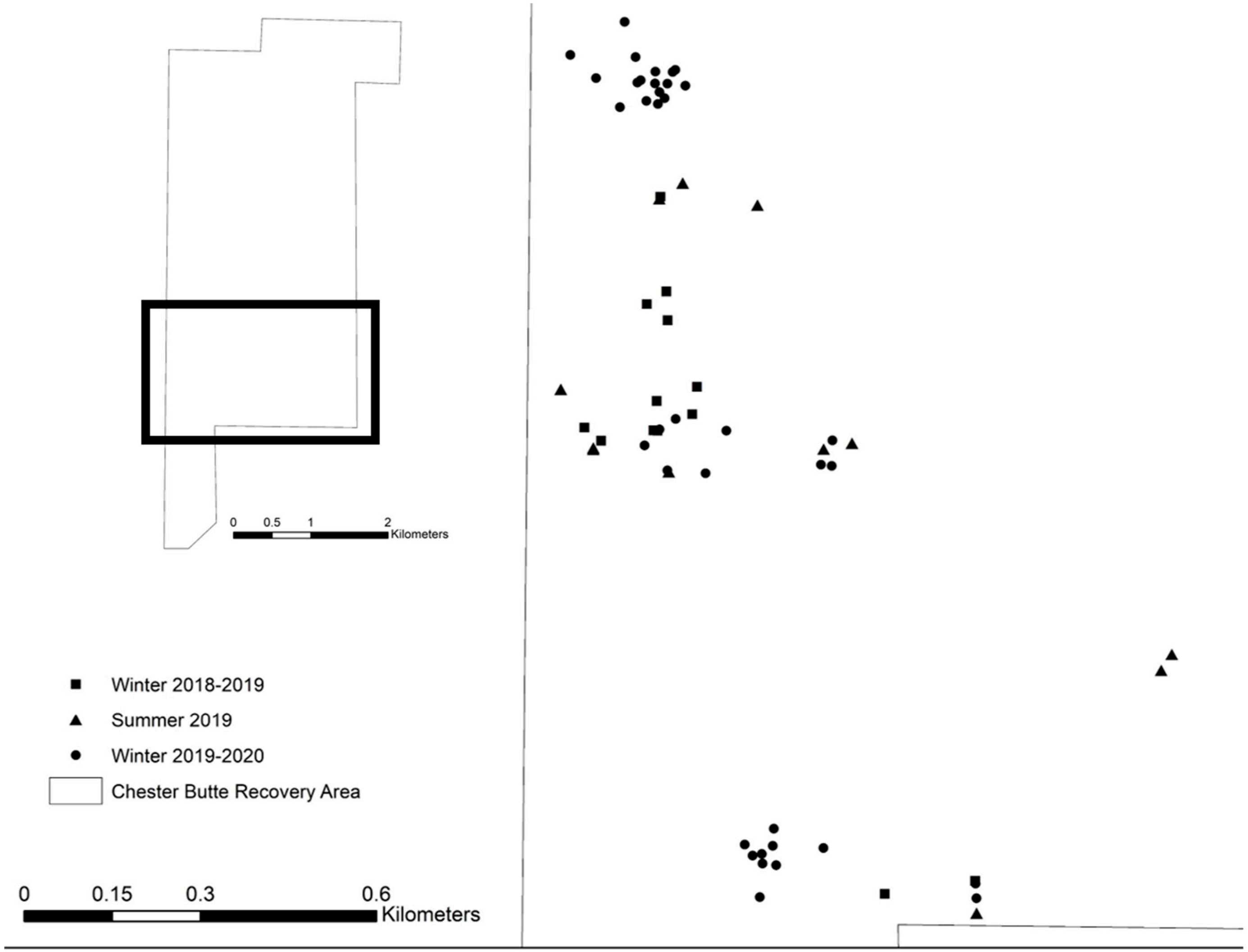

Figure S2. Location of active pygmy rabbit (*Brachylagus idahoensis*) burrows across the habitat identified during monitoring surveys in the Chester Butte recovery area, in central Washington state, during winter 2018-2019 (■), summer 2019 (▲), and winter 2019-2020 (•).

Table S1. AICc values, △AICc, model weights, cumulative model weights, and log-likelihood values at the 95% confidence interval of the top models describing apparent survival rate of juvenile pygmy rabbits (*Brachylagus idahoensis*) after reintroduction into Sagebrush Flat/CRP sits in central Washington, USA, from 2012-2016. See Figure 7 for relationships between survival and significant variables in the top model. Year refers to the release year and was included in all models because it was highly significant. Day represents the release day based on Julian calendar, and weight represents the weight (g) of a juvenile at time of release. Columbia Basin ancestry (CB Ancestry) was a genetic estimate based on program STRUCTURE. Homozygosity was determined from genotypes of individuals in R-package GENHET. Only models that that performed better than the intercept only (due to random factors) were included.

Model	n	Variables	AIC	△AIC	wi	∑wi	Log-Likelihood
Juvenile Survival - Sagebrush Flat/CRP	1660	Year + Day + Weight + Homozygosity	781.33	0	0.43	0.43	-382.62
		Year + Day + Weight + Sex + Homozygosity	783.35	2.02	0.16	0.59	-382.62
		Year + Weight + Homozygosity	784.90	3.57	0.07	0.67	-385.42
		Year + Day + Weight + Sex + CB Ancestry + Homozygosity	784.99	3.66	0.07	0.74	-382.43
		Year + Day + Weight	785.02	3.69	0.07	0.80	-385.48
		Year + Day + Weight + CB Ancestry	786.68	5.35	0.03	0.83	-385.30
		Year + Weight + CB Ancestry + Homozygosity	786.78	5.45	0.03	0.86	-385.35
		Year + Weight + Sex + Homozygosity	786.85	5.52	0.03	0.89	-385.38
		Year + Day + Weight + Sex	787.04	5.71	0.03	0.92	-385.48
		Year + Day + Homozygosity	788.23	6.90	0.01	0.93	-387.08
		Year + Weight	788.50	7.17	0.01	0.94	-388.23
		Year + Day + CB Ancestry + Homozygosity	788.58	7.25	0.01	0.95	-386.25
		Year + Day + Weight + Sex + CB Ancestry	788.70	7.37	0.01	0.96	-385.29
		Year + Weight + Sex + CB Ancestry + Homozygosity	788.74	7.41	0.01	0.97	-385.31
		Year + Day + Sex + Homozygosity	790.15	8.82	0.01	0.98	-387.03
		Year + Weight + CB Ancestry	790.39	9.06	0.00	0.98	-388.16
		Year + Weight + Sex	790.45	9.12	0.00	0.99	-388.19
		Year + Day + Sex + CB Ancestry + Homozygosity	790.53	9.20	0.00	0.99	-386.21
		Year + Day	792.24	10.91	0.00	1.00	-390.09
		Year + Weight + Sex + CB Ancestry	792.34	11.01	0.00	1.00	-388.13
		Year + Day + CB Ancestry	792.62	11.29	0.00	1.00	-389.28
		Year + Day + Sex	794.16	12.83	0.00	1.00	-390.04
		Year + Day + Sex + CB Ancestry	794.57	13.24	0.00	1.00	-389.24
		Year + Homozygosity	805.38	24.05	0.00	1.00	-396.66
		Year + CB Ancestry + Homozygosity	805.61	24.28	0.00	1.00	-395.77
		Year + Sex + Homozygosity	807.37	26.05	0.00	1.00	-396.65
		Year + Sex + CB Ancestry + Homozygosity	807.62	26.29	0.00	1.00	-395.76
		Year	809.50	28.17	0.00	1.00	-399.73
		Year + CB Ancestry	809.75	28.42	0.00	1.00	-398.85
		Year + Sex + CB Ancestry	811.51	30.18	0.00	1.00	-399.73
		Year + Sex + CB Ancestry	811.76	30.43	0.00	1.00	-398.85
		Intercept Only	957.49	176.16	0.00	1.00	-477.74

Table S2. AICc values, △AICc, model weights, cumulative model weights, and log-likelihood values at the 95% confidence interval of the top models describing apparent adult survival rate of adult pygmy rabbits (*Brachylagus idahoensis*) after reintroduction into Sagebrush Flat/CRP sits in central Washington (2012-2016). See Figure 6 for the top model. Year was not included in the model because the number of released adults each year varied greatly. Day represents the release day based on Julian calendar, and weight represents the weight (g) of an adult at time of release. Columbia Basin ancestry (CB Ancestry) were genetic estimates based on program STRUCTURE. Homozygosity was determined from genotypes of individuals in R-package GENHET. Only models that that performed better than the intercept only (due to random factors) were included.

Model	n	Variables	AIC	△AIC	wi	∑wi	Log-Likelihood
Adult Survival -	177	Day	144.36	0.00	0.20	0.20	-70.15
Sagebrush Flat/CRP		Day + Homozygosity	144.73	0.37	0.17	0.37	-69.30
		Day + CB Ancestry	145.83	1.47	0.10	0.46	-69.84
		Day + CB Ancestry + Homozygosity	145.84	1.48	0.10	0.56	-68.81
		Day + Sex	146.37	2.01	0.07	0.63	-70.12
		Day + Weight	146.41	2.05	0.07	0.70	-70.13
		Day + Sex + Homozygosity	146.66	2.29	0.06	0.77	-69.21
		Day + Weight + Homozygosity	146.75	2.39	0.06	0.83	-69.26
		Day + Sex + CB Ancestry + Homozygosity	147.91	3.55	0.03	0.86	-68.78
		Day + Sex + CB Ancestry	147.91	3.55	0.03	0.89	-69.84
		Day + Weight + CB Ancestry	147.92	3.56	0.03	0.93	-69.84
		Day + Weight + Sex	148.46	4.10	0.03	0.95	-70.12
		Day + Weight + Sex + Homozygosity	148.76	4.40	0.02	0.98	-69.21
		Day + Weight+ Sex + CB Ancestry	150.03	5.67	0.01	0.99	-69.84
		Day + Weight + Sex + CB Ancestry + Homozygosity	150.05	5.69	0.01	1.00	-68.78
		Homozygosity	158.98	14.62	0.00	1.00	-77.45
		Intercept Only	159.90	15.54	0.00	1.00	-78.94
